# Colonic Perineurioma’s Malignant Proximity to Serrated Colonic Polyps

**DOI:** 10.7759/cureus.4815

**Published:** 2019-06-03

**Authors:** Sindhura Kolli, Srilaxmi Gujjula, Mel A Ona

**Affiliations:** 1 Internal Medicine, The Brooklyn Hospital Center, Affiliate of the Mount Sinai Hospital, Brooklyn, USA; 2 Gastroenterology, Pali Momi Medical Center, Honolulu, USA

**Keywords:** perineurioma, serrated polyp, colonic polyp, colon polyp, braf, gist

## Abstract

A colonic perineurioma is often considered a benign cousin to a colonic polyp. However, in the submucosal type of perineurioma, it is important to rule out the malignant gastrointestinal stromal tumor (GIST). Alternatively, in the BRAF-positive serrated types of perineuriomas, surveillance is equivalent to intervals designated to serrated polyps due to a similar malignant potential. These versions serve as reminders that colonic perineuriomas are not to be disregarded.

## Introduction

This case examines the epidemiology, classification, clinical features, diagnosis, treatment, and most importantly, the differential diagnoses of a colonic perineurioma. Heeding the malignant potential of its variants and possible differentials influences the course of treatment, recurrence, and surveillance which is vital for a gastroenterologist.

## Case presentation

**Figure 1:**
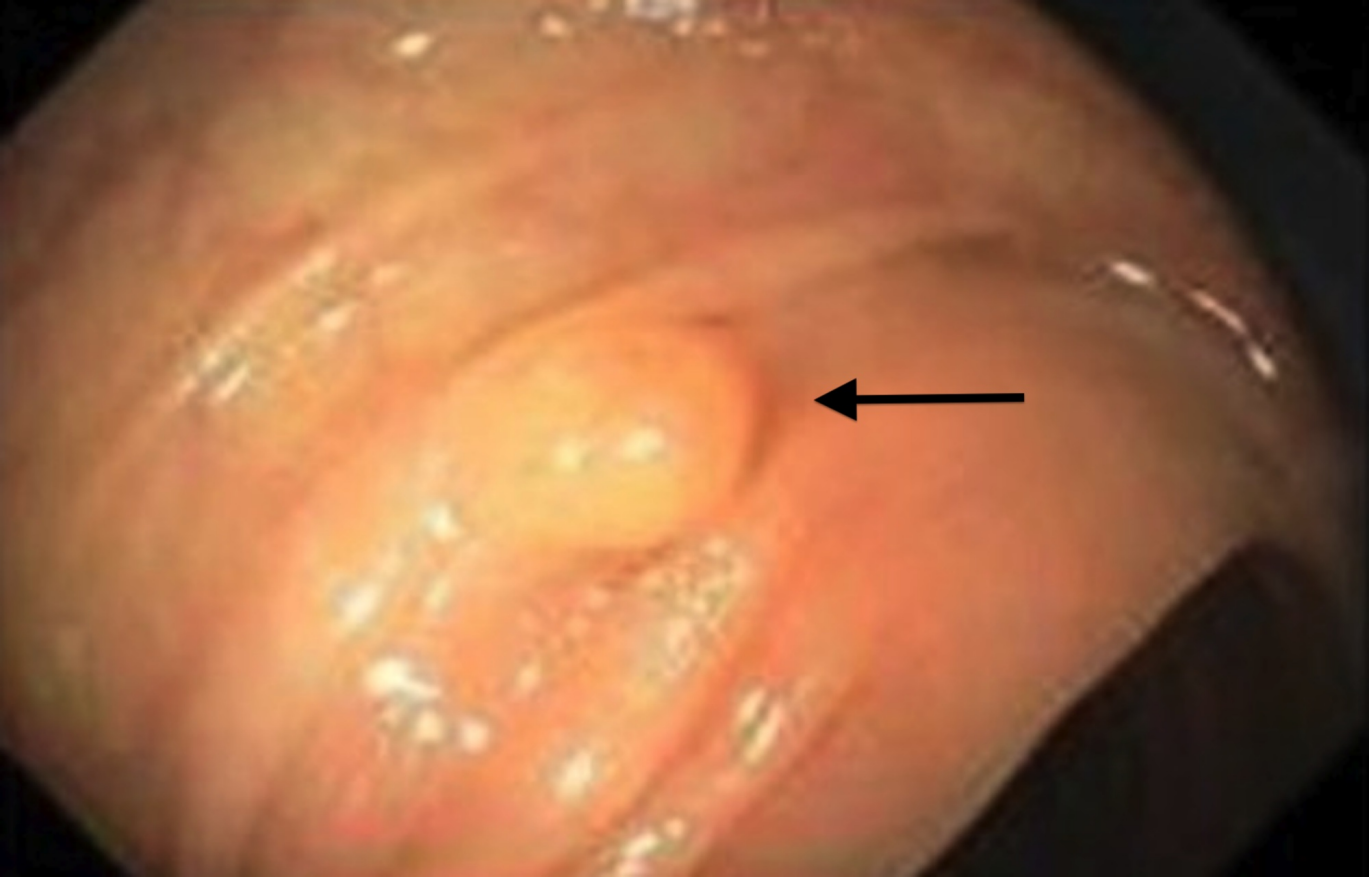
Polyp in the transverse colon

A 35-year-old Caucasian female presented with sharp, non-radiating, epigastric abdominal pain associated with alternating constipation and diarrhea. A computed tomography (CT) scan demonstrated a thickened stomach wall and diverticulitis at the splenic flexure. Vital signs and labs were within normal limits. Colonoscopy showed a unusual looking polyp in the transverse colon (Figure 1).

**Figure 2:**
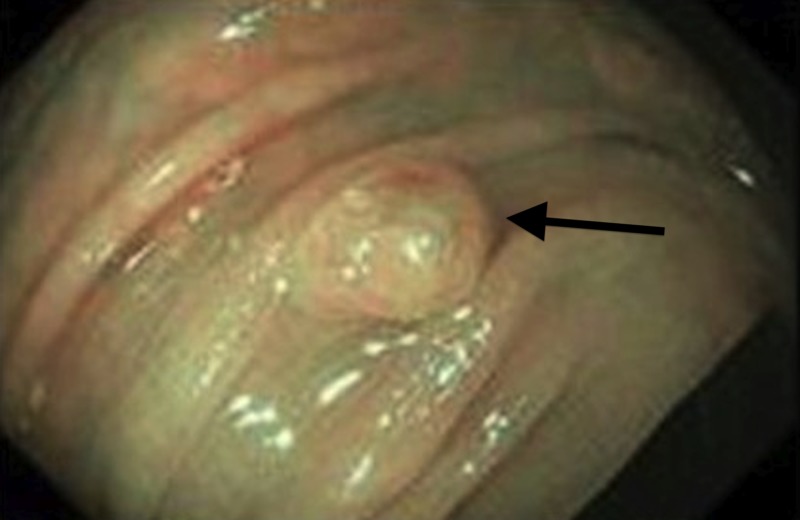
NBI showing unusual vessel appearance and surface pit pattern NBI, narrow band imaging

Narrow band imaging (NBI) did not demonstrate typical vessel appearance or polyp pit pattern (Figure 2). No other abnormalities were observed. Biopsies revealed a perineurioma. The patient was treated conservatively, her pain resolved, and she was discharged with plan for follow-up at recommended surveillance.

## Discussion

Colonic perineuriomas are rare benign mucosal lesions with a serrated or hyperplastic architecture composed of perineural cells. They are usually discovered incidentally during screening colonoscopies. With an incidence of 0.1% to 0.46%, they are found distal to the splenic flexure in the sigmoid colon and the rectum mostly as polyps and rarely as solitary masses. No clear pathogenesis has been found. These polyps are considered benign reactive processes; however, the serrated variant confers malignant potential from BRAF mutations resulting in fibroblast differentiation and proliferation [1].

Mucosal perineuriomas are asymptomatic in comparison to the submucosal or intramural masses. Morphology alone can lead to strong suspicion of perineurioma, but the expression of at least two perineural markers, such as glucose transporter-1 (GLUT-1), Claudin-1, and epithelial membrane antigen (EMA), are generally required to confirm diagnosis [2]. Even though EMA stains are dependent on the antibody dilution and can be weak, Claudin-1 and GLUT-1 are considered to have high sensitivity and specificity to mark perineurial differentiation [3]. The mean age at diagnosis was 60-years-old with a slight female majority [2].

Differential diagnosis is dependent on the origin of the lesion: intramucosal or submucosal. Neurofibroma, ganglioneuroma, schwannoma, neuroma, Schwann cell hamartoma, benign epithelioid nerve sheath tumor, and leiomyoma of the muscularis mucosae should be considered for intramucosal colorectal perineuriomas. For submucosal perineuriomas, schwannomas and the only malignant differential, a gastrointestinal stromal tumor (GIST) must be excluded [3]. Colorectal perineuriomas are considered to be benign. However, due to the malignancy potential in BRAF-positive serrated variants of colorectal perineurioma, we suggest abbreviated postpolypectomy surveillance repeat screening at similar intervals as serrated colorectal polyps [1].

## Conclusions

A colonic perineurioma is considered a benign process. However, it is vital for gastroenterologists and patients to be aware of the malignant potential in BRAF-positive serrated variants and to rule out malignant differentials, such as GISTs, in submucosal perineuriomas.
